# Bacterial Flagellin Triggers Cardiac Innate Immune Responses and Acute Contractile Dysfunction

**DOI:** 10.1371/journal.pone.0012687

**Published:** 2010-09-13

**Authors:** Joelle Rolli, Nathalie Rosenblatt-Velin, Jianhui Li, Noureddine Loukili, Sandra Levrand, Pal Pacher, Bernard Waeber, François Feihl, Patrick Ruchat, Lucas Liaudet

**Affiliations:** 1 Department of Intensive Care Medicine, University Hospital Medical Center and Faculty of Biology and Medicine, Lausanne, Switzerland; 2 Division of Pathophysiology, University Hospital Medical Center and Faculty of Biology and Medicine, Lausanne, Switzerland; 3 Department of Cardiovascular Surgery, University Hospital Medical Center and Faculty of Biology and Medicine, Lausanne, Switzerland; 4 Laboratory of Physiologic Studies, National Institutes of Health/National Institute on Alcohol Abuse and Alcoholism, Bethesda, Maryland, United States of America; 5 Department of Hepatobiliary Surgery, the First Affiliated Hospital, College of Medicine, Zhejiang University, Hangzhou, China; Institut Pasteur, France

## Abstract

**Background:**

Myocardial contractile failure in septic shock may develop following direct interactions, within the heart itself, between molecular motifs released by pathogens and their specific receptors, notably those belonging to the toll-like receptor (TLR) family. Here, we determined the ability of bacterial flagellin, the ligand of mammalian TLR5, to trigger myocardial inflammation and contractile dysfunction.

**Methodology/Principal Findings:**

TLR5 expression was determined in H9c2 cardiac myoblasts, in primary rat cardiomyocytes, and in whole heart extracts from rodents and humans. The ability of flagellin to activate pro-inflammatory signaling pathways (NF-kappaB and MAP kinases) and the expression of inflammatory cytokines was investigated in H9c2 cells, and, in part, in primary cardiomyocytes, as well as in the mouse myocardium in vivo. The influence of flagellin on left ventricular function was evaluated in mice by a conductance pressure-volume catheter. Cardiomyoyctes and intact myocardium disclosed significant TLR5 expression. In vitro, flagellin activated NF-kappaB, MAP kinases, and the transcription of inflammatory genes. In vivo, flagellin induced cardiac activation of NF-kappaB, expression of inflammatory cytokines (TNF alpha, IL-1 beta, IL-6, MIP-2 and MCP-1), and provoked a state of reversible myocardial dysfunction, characterized by cardiac dilation, reduced ejection fraction, and decreased end-systolic elastance.

**Conclusion/Significance:**

These results are the first to indicate that flagellin has the ability to trigger cardiac innate immune responses and to acutely depress myocardial contractility.

## Introduction

Septic shock, characterized by systemic inflammation and cardiovascular collapse, is triggered upon the recognition of pathogen-associated molecular patterns (PAMPs) by pattern recognition receptors (PRRs) [1]. Toll-like Receptors (TLRs) represent a major family of PRRs, playing a front-line role in host defenses by inducing innate immune responses through nuclear factor kappa B (NF-κB) signaling [2], as well as through members of the mitogen-activated protein kinase (MAPK) family [3], [4].

One of the vital organs adversely affected in septic shock is the heart [5]. Myocardial contractile failure occurs in 24 to 44% of patients with septic shock [6]. Its pathogenic mechanisms remain largely unknown, although it is established that pro-inflammatory cytokines such as TNFα and IL-1β are potent cardiodepressant effectors [7]. Recent findings indicate that the heart expresses several TLRs, implying that it may sense bacterial PAMPs to mount its own immune response and promote its own dysfunction [8]. Indeed, studies reported that lipopolysaccharide (LPS) from Gram-negative bacteria induced myocardial inflammation and cardiac dysfunction by interacting with its ligands, CD14 and TLR4, on cardiomyocytes [9], [10]. The relevance of these results is, however, questionable, as they were obtained with very high doses of LPS (25 mg/kg) and relied on indirect, load-dependent, indices of cardiac contractility. In fact, several animal studies using load-independent measurements of cardiac contractiliy could not reproduce such direct negative inotropic effects of LPS [11], [12]. Beside LPS, most Gram-negative bacteria also release substantial amounts of flagellin, the main structural protein of the bacterial flagellum [13]. Flagellin elicits immune responses upon binding to its receptor, TLR5 [14], expressed by immune and various epithelial cells [13], [15], [16], induces systemic inflammation in rodents [13], [14], [17], and circulates in significant concentrations in the plasma of septic shock patients [18]. Given these different properties, we hypothesized that flagellin might trigger innate immune responses in the heart and represent a potential mediator of myocardial contractile dysfunction in sepsis.

## Results

### Cardiomyocytes express TLR5

**Figure 1:**
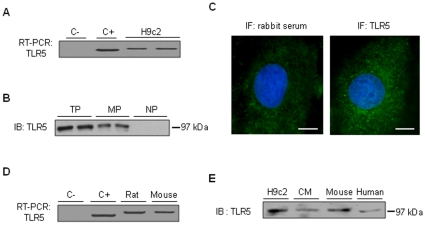
Expression of TLR5 by cultured cardiomyocytes and whole heart tissue. A, RT-PCR analysis of *tlr5* transcription on H9c2 total RNA. C-, negative control (no RNA); C+, positive control. Sizes of the amplified fragments: 310 bp (C+); 381 bp (TLR)5. B, TLR5 protein expression in H9c2 cells. Total proteins (TP), membrane proteins (MP), nuclear proteins (NP). C, Immunofluorescence detected the presence of TLR5 in H9c2 cells. Normal rabbit serum (left) instead of anti-TLR5 antibody for negative control. Scale bar size: 10 µm. D, RT-PCR analysis of *tlr5* transcription in rat and mouse heart. E, Constitutive expression of TLR5 protein in H9c2, rat primary ventricular cardiomyocytes (CM), and mouse and human heart.

In H9c2 cells, we detected both TLR5 mRNA (Fig. 1A) and TLR5 protein (Fig. 1B and C), migrating at approximately 100 kDa (reported size of TLR5: 97 kDa [19]). TLR5 mRNA was detected in rat and mouse hearts (Fig. 1D), and TLR5 protein was also expressed in primary ventricular cardiomyocytes, and in mouse and human hearts (Fig. 1E).

### Flagellin activates NF-κB in cultured cardiomyocytes

**Figure 2:**
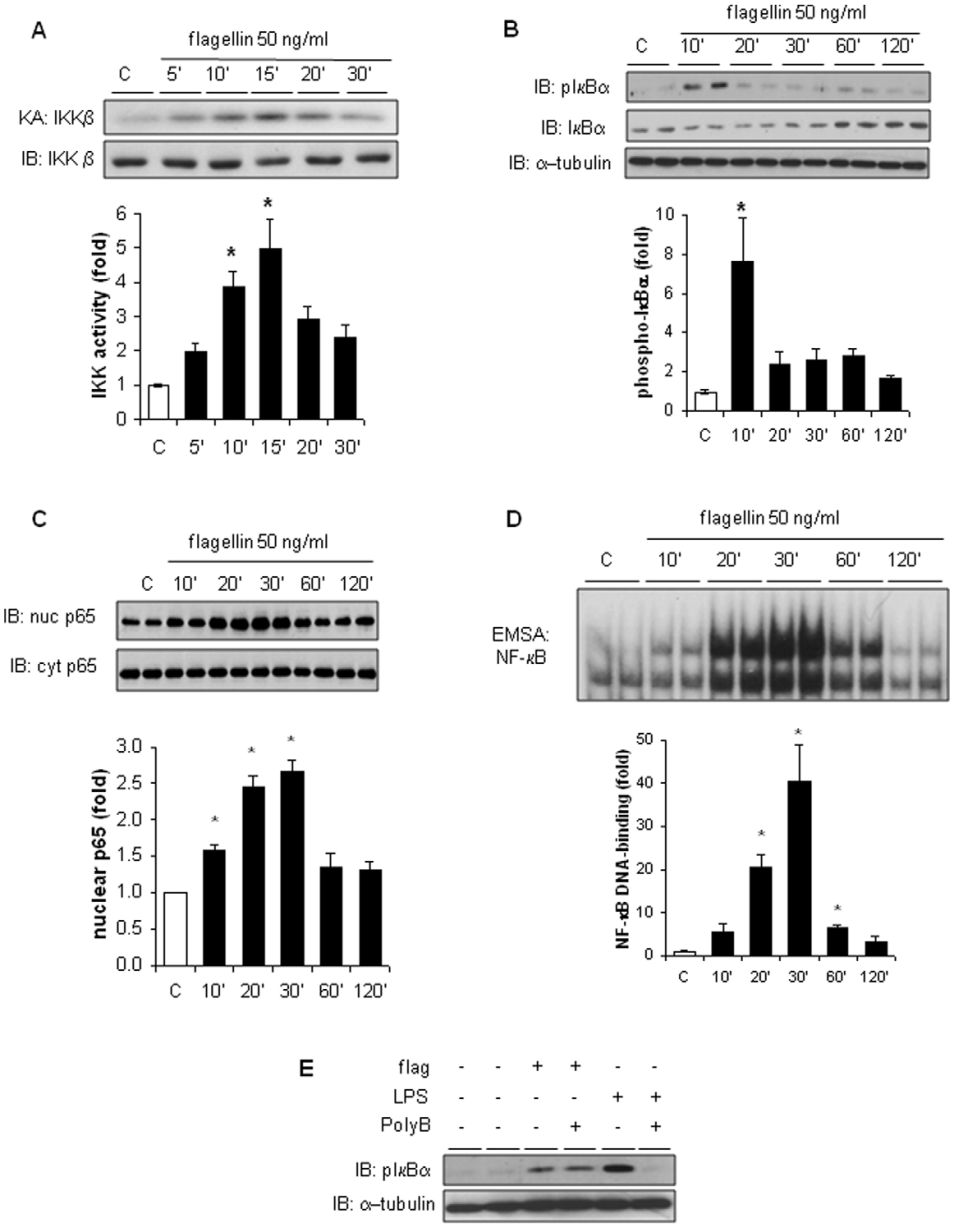
Flagellin activates the IKK/NF-κB signaling pathway in H9C2 cardiomyocytes. H9c2 cells were unstimulated (control, C) or stimulated with flagellin (50 ng/ml) for 5 to 120 minutes. A, IKK Kinase assay (KA) showing increased IKK activity after flagellin, starting at 10 minutes. IKKβ immunoblot (IB), loading control. B–C, IκBα phosphorylation and degradation, and p65 nuclear translocation are elicited by flagellin. α-tubulin, loading control. D, Increased NF-κB-DNA binding activity elicited by flagellin, peaking at 30 minutes. E, Polymyxin B did not block IκBα phosphorylation by flagellin but blocked that triggered by LPS. α-tubulin, loading control. * p<0.05 versus control C (ANOVA followed by Dunnett test).

The engagement of TLR5 upon flagellin binding initiates a signaling cascade leading to the activation of NF-κB [13]. TLR signaling activates the upstream inhibitor of NF-κB kinase (IKK), which induces phosphorylation and degradation of the inhibitor IκBα, allowing NF-κB nuclear translocation [20]. In H9c2 cells, flagellin induced transient activation of IKK peaking at 15 minutes (Fig. 2A), associated with phosphorylation and degradation of IκBα (Fig. 2B), followed by IκBα resynthesis after 60 minutes for negative feedback [21]. NF-κB activation was confirmed by the nuclear translocation of NF-κB p65 subunit (Fig. 2C) and NF-κB -DNA binding (Fig. 2D). These effects of flagellin could not be related to any LPS contamination, since LPS concentration in the flagellin preparation measured by the Limulus assay was negligible (<15 pg LPS/50 ng flagellin) and since the LPS binding agent polymyxin B did not affect IκBα phosphorylation induced by flagellin (Fig. 2E).

**Figure 3:**
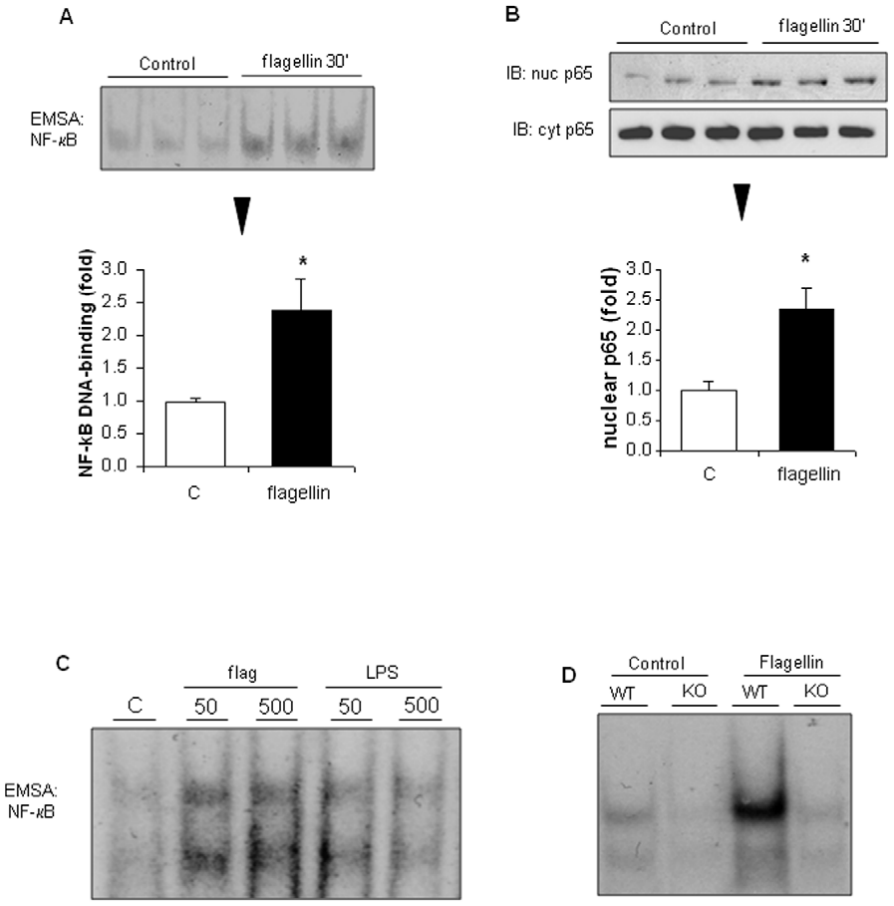
Flagellin activates NF-κB in primary murine cardiomyocytes via TLR5. A–B, Nuclear translocation of p65 and NF-κB-DNA binding triggered by flagellin in primary rat ventricular myocytes after 30 minutes. C, Concentration-response experiments of NF-κB-DNA binding in primary rat cardiomyocytes exposed either to flagellin or LPS (both at 50 ng/ml or 500 ng/ml) for 30 minutes. * p<0.05 versus control C (t test).

In primary rat ventricular cardiomyocytes, flagellin (50 ng/ml) also induced nuclear p65 translocation and NF-κB-DNA binding (Fig. 3A and B). There was no further increase in NF-κB-DNA binding at higher flagellin concentrations (up to 500 ng/ml) (Fig. 3C). NF-κB activation produced by LPS at similar concentrations (50–500 ng/ml) was less pronounced than with flagellin. The activation of NF-κB by flagellin was entirely dependent on an intact TLR5-dependent signaling, as indicated by the lack of NF-κB activation in TLR5 KO cardiomyocytes, contrasting with a strong activation in WT cardiomyocytes (Fig. 3D)

### Flagellin induces transcription of NF-κB-dependent genes in H9c2 cells

**Figure 4:**
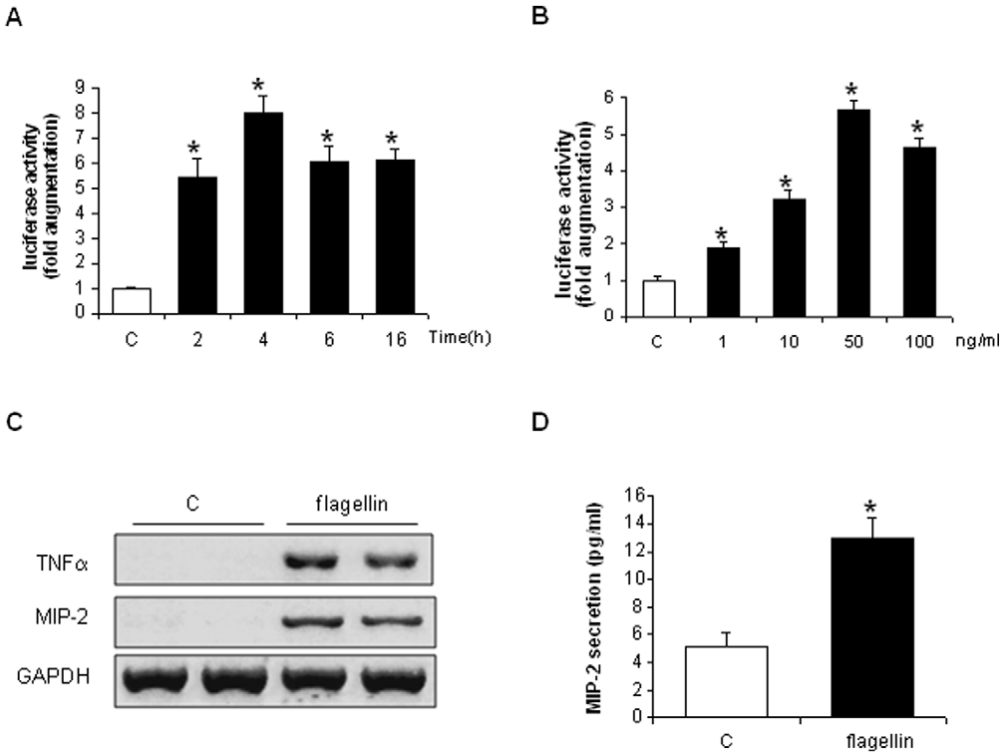
Transcription of NF-κB-dependent genes in H9c2 cells after flagellin challenge. A, Time-course experiments. Luciferase activity was maximally induced after 2 to 4 hours of flagellin (50 ng/ml). B, Dose-response experiments. Luciferase activity was slightly induced by 1 ng/ml flagellin and maximally induced by 50 ng/ml flagellin (4 hours). C, Transcript levels of TNFα and MIP-2 mRNA (RT-PCR) in unstimulated cells (control, C) and after flagellin (50 ng/ml, 1 hour). GAPDH, control. D, Flagellin induced synthesis and secretion of MIP-2 by H9C2 cells (ELISA). * p<0.05 versus control C (ANOVA followed by Dunnett test A and B; *t*-test, D).

Flagellin increased the transcription of a transfected NF-κB-dependent luciferase gene in H9c2 cells (Fig. 4A), with a threshold inducing concentration of only 1 ng/ml (Fig. 4B), and also promoted the transcription of the NF-κB-dependent genes TNFα and MIP-2 (Fig. 4C), the latter being secreted in H9c2 supernatants (Fig. 4D).

### Flagellin activates MAPK signaling pathway in H9c2 cells

**Figure 5:**
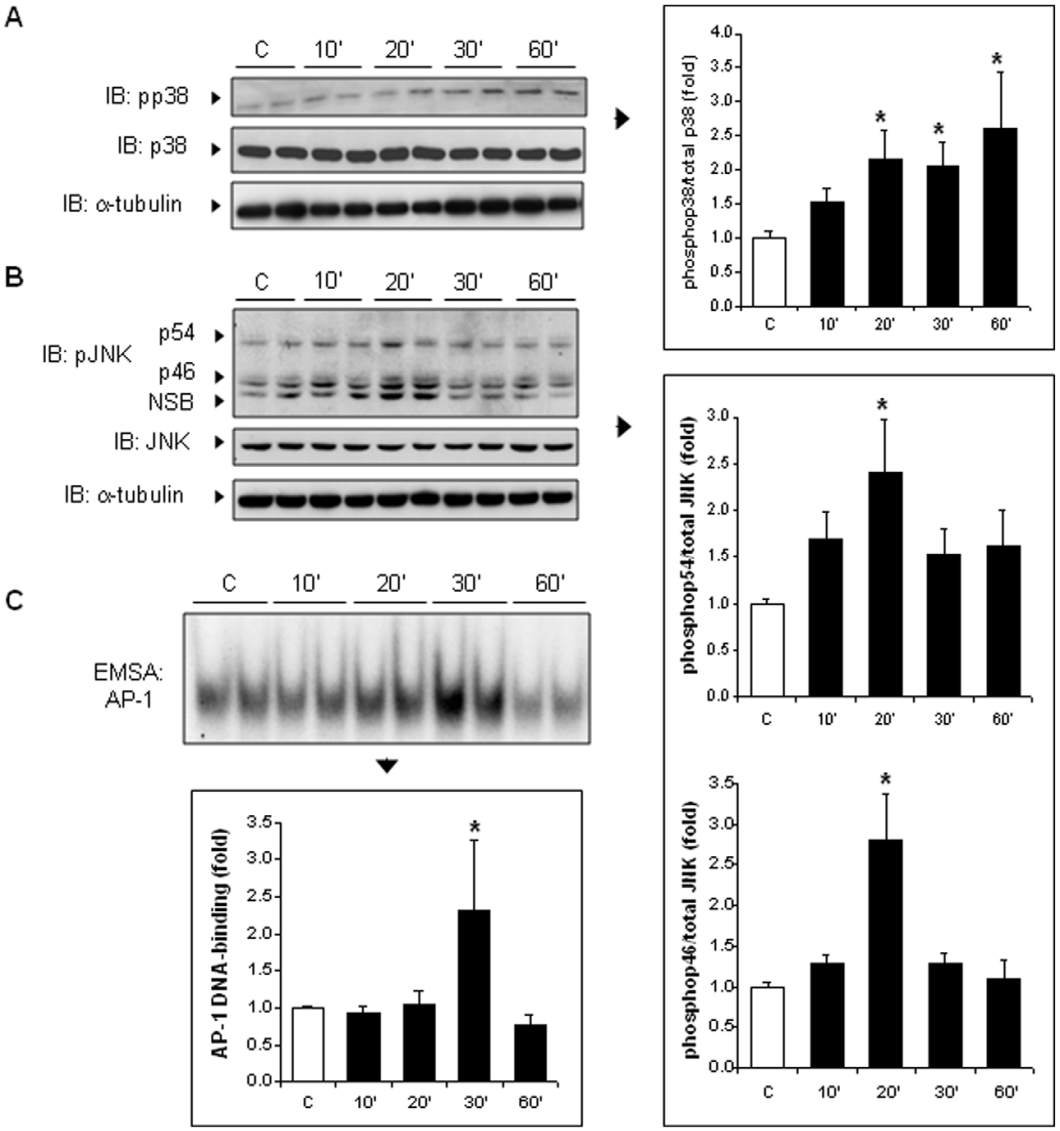
Flagellin activates MAP kinase signaling in H9c2 cells. A, Flagellin induced p38 phosphorylation from 20 to 60 minutes. Total p38 remained unchanged. B, Phosphorylation of JNK was triggered by flagellin after 20 minutes. Total JNK1 remained unchanged. C, Flagellin triggered AP-1-DNA binding activity with a peak at 30 minutes. NSB: non-specific band. * p<0.05 versus control C (ANOVA followed by Dunnett test).

Since the interaction of PAMPs with TLRs also triggers activation of the mitogen-activated protein kinases p38 and c-Jun NH_2_-terminal kinase (JNKs) [3], [4], the effects of flagellin on this signaling cascade was evaluated. As shown in Fig. 5A and B, flagellin activated the phosphorylation of both p38 and JNK in H9c2 cells, and also enhanced the binding of AP-1 to its DNA-response element, known to be activated by JNK (Fig. 5C).

### Flagellin activates NF-κB and pro-inflammatory signaling in the mouse heart in vivo, but does not trigger significant myocardial cell death

**Figure 6:**
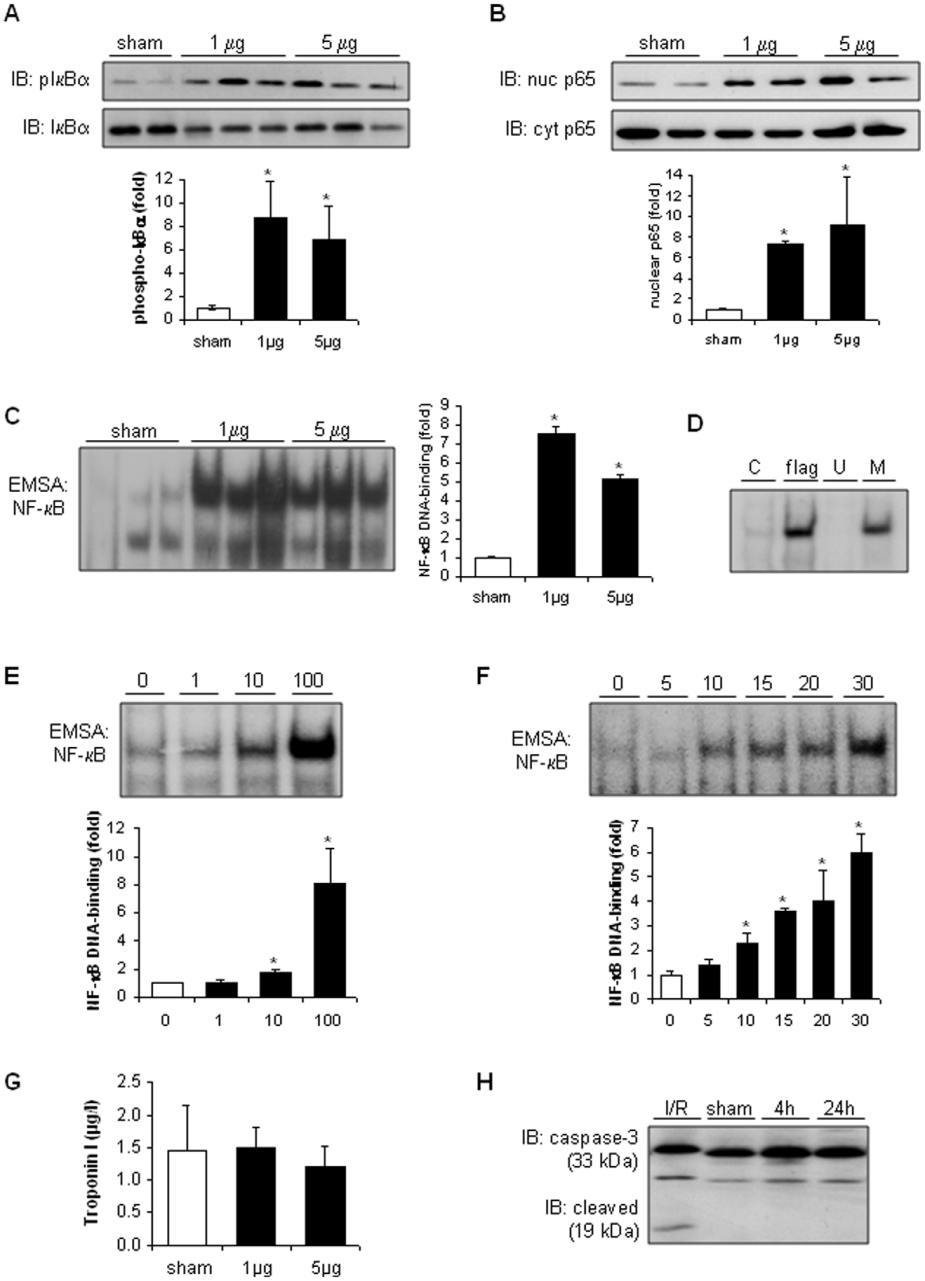
Flagellin activates NF-κB in the heart and does not induce myocardial cell death in vivo. A–B, Flagellin increased phosphorylation and degradation of IκBα, and nuclear p65 trasnlocation after 30 minutes in myocardial extracts. No differences were detected between the two doses of flagellin (1 and 5 µg). C, Flagellin (1 and 5 µg) induced marked NF-κB-DNA binding activity in heart tissue after 30 minutes. D, Specificity of the NF-κB-DNA binding. The band shift disappears in the presence of an excess of cold unmutated (U), but not mutated (M), probe. E, Dose-response experiments. NF-κB-DNA binding activity (30 min after flagellin) significantly increased at 10 ng and further increased at 100 ng/mouse. F, Time-course experiments. NF-κB-DNA binding induced by flagellin (1 or 5 µg) started to increase after 10 minutes. G, Cardiac troponin I was not increased 4 hours after flagellin (Immunoassay). H, Myocardial cleavage of caspase-3 was strongly induced by 30 minutes ischemia and 3 hours reperfusion (I/R, positive control), but was not detectable 4 to 24 hours after flagellin (1 µg). * p<0.05 versus control C (ANOVA followed by Dunnett test).

Injection of 1 or 5 µg (respectively 40 or 200 µg/kg) flagellin to BALB/c mice robustly induced IκBα phosphorylation and degradation (Fig. 6A), p65 nuclear translocation (Fig. 6B) and NF-κB-DNA binding (Fig. 6C) in whole heart within 30 minutes. Specificity of the NF-κB-DNA binding was ascertained by cold competition experiments (Fig. 6D). The threshold NF-κB-inducing dose of flagellin was 10 ng per mouse (400 ng/kg; Fig. 6E). In time-course experiments, NF-κB was activated in the heart as early as 10 minutes after the injection of flagellin (Fig. 6F).

Flagellin did not induce myocardial cell death (necrotic or apoptotic), as indicated by the lack of increase of serum troponin (Fig. 6G) and the lack of cleavage of caspase-3 (Fig. 6H), the main executioner apoptotic caspase, up to 24 hours after the injection of flagellin. As a positive control, cardiac tissue was obtained following myocardial ischemia-reperfusion in rats, which strongly activates caspase-3 cleavage [22].

**Figure 7:**
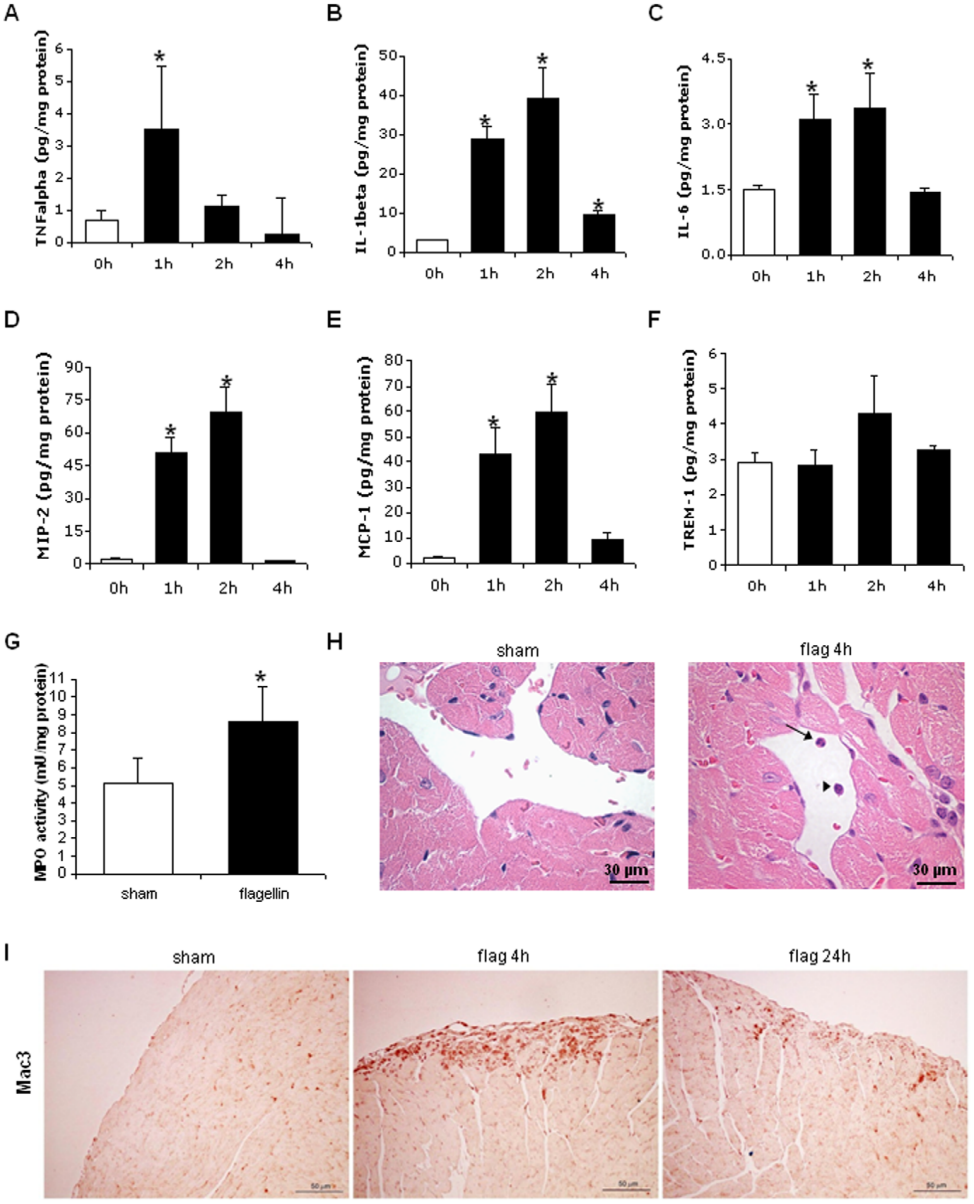
Flagellin triggers the generation of pro-inflammatory cytokines in the mouse heart in vivo. Flagellin (1 µg) induced significant increases of TNF-, IL-1, IL-6, MIP-2 and MCP-1 (A–E), but not soluble TREM-1 (F) in whole hearts from BALB/c mice (n = 5 mice per condition). Flagellin also induced a modest, but significant increase of myeloperoxydase (MPO) activity in the heart after 4 h (G). Histologically, rare polymorphonuclear (arrow) and mononuclear (arrowhead) leukocytes were found within cardiac blood vessels (H). Immunohistochemical studies revealed a slight recruitment of Mac-3 positive mononuclear cells, located in the subepicardial region (I). * p<0.05 versus control C (ANOVA followed by Dunnett test A to F; *t*-test, G). Histological pictures are representative of 3 mice/group. Scale bar size: 30 µm (H); 50 µm (I).

Flagellin induced significant cardiac expression of TNFα, IL-1β, IL-6, MIP-2 and MCP-1 (Fig. 7A–E). This was associated with modest cardiac recruitment of leukocytes, as evidenced by a slightly increased activity of myeloperoxidase (Fig. 7G), the presence of rare polymorphonuclear cells (PMN) in cardiac blood vessels after 4 hours (Fig. 7H), and an increased Mac-3 immunoreactivity in the subepicardial region after 4 and 24 h (Fig. 7I), pointing to some infiltrating mononuclear cells. The degree of activation of these cells appeared limited, as supported by the lack of increase of soluble TREM-1, a biomarker of myeloid cell activation [23], in cardiac tissue (Fig. 7F).

**Figure 8:**
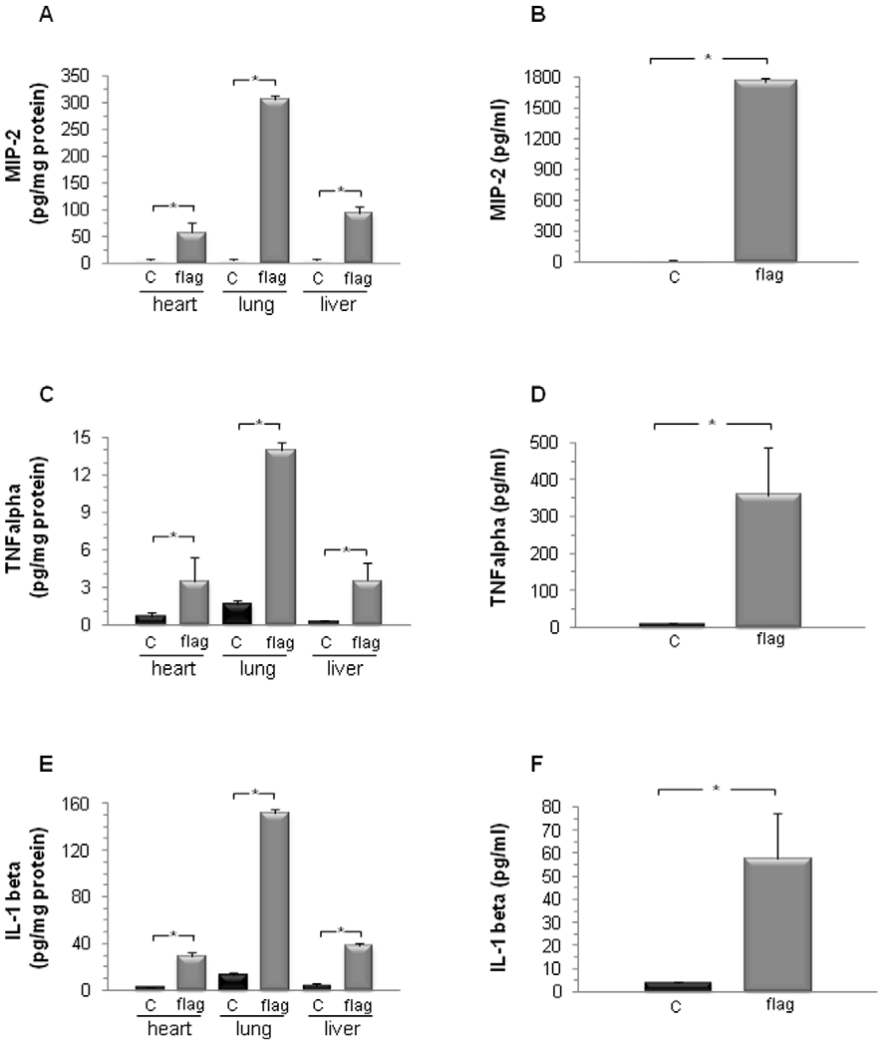
Flagellin induces widespread generation of pro-inflammatory cytokines. The levels of MIP-2 (A, B), TNFα (C, D) and IL-1β (E, F) were determined by ELISA in the lung and liver (left panel) as well as in plasma (right panel) in control (C) and 1 h after systemic flagellin administration (n = 3mice/group). A significant increase of these cytokines was detected both in organs and in plasma. *p<0.05 vs control C (t test).

The inflammatory response triggered by flagellin was not restricted to the heart, as indicated by marked elevations of TNFα, IL-1β, and MIP-2 also noted in the lung and liver, as well as a significant increase of circulating cytokines, as measured 1 h after the administration of flagellin (Fig. 8), in line with other reports on the effects of flagellin at the systemic level [13], [17]. The cytokine response of the heart was comparable to that of the liver, but was less pronounced than that of the lung, which agrees with previous findings on flagellin-induced pulmonary inflammation [18].

### Flagellin impairs cardiac function in vivo

**Figure 9:**
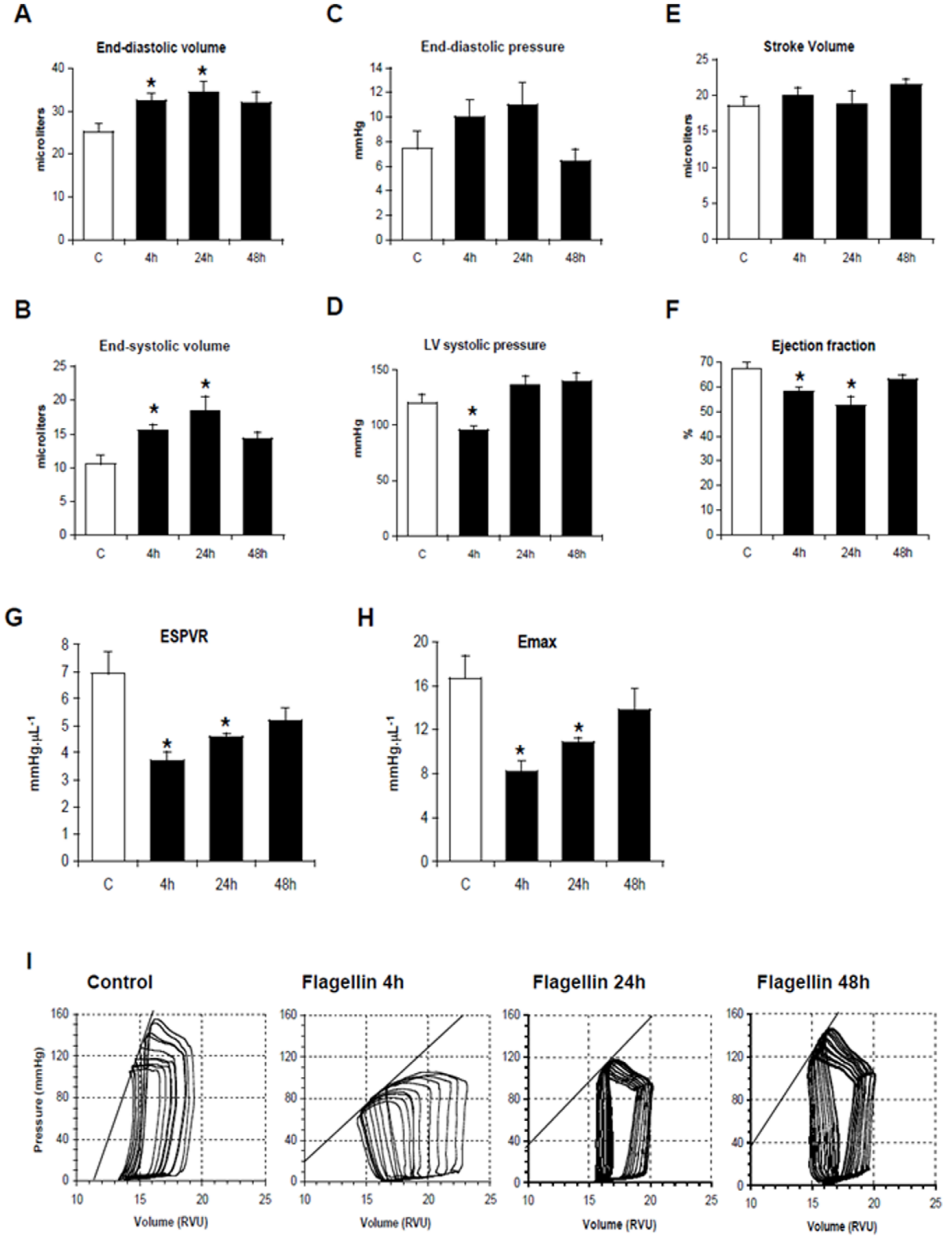
Flagellin transiently impairs cardiac function in anesthetized mice. A–B, Flagellin (1 µg) induced cardiac dilation at 4 and 24 h, but not 48 h, as shown by increased end-systolic and end-diastolic LV volumes. C–D, Flagellin significantly reduced LV maximal systolic pressure at 4 h and tended to increase LV end-diastolic pressure at 4 and 24 h. E–F, Flagellin did not induce changes of stroke volume, but significantly reduced LV ejection fraction at 4 and 24 h, but not 48 h. G–H, The slope of the ESPVR (Ees), as well as maximal elastance (Emax), were significantly reduced by flagellin, at 4 and 24 h, but not 48 h. I, Representative examples of end-systolic PV relationships (ESPVR) in control (C) and flagellin-treated mice at 4, 24 and 48 h. RVU: Relative Volume Units. * p<0.05 versus control C (ANOVA followed by Dunnett test; n = 5–7 mice/group).

Flagellin induced, after 4 and 24 hours, significant increases in end-systolic and end-diastolic LV volumes (Fig. 9A and B). LV systolic pressure significantly decreased only at 4 hours, and there was a trend towards increased LV end-diastolic pressure at 4 and 24 hours (Fig. 9C and D). There was a significant reduction in ejection fraction, with no change in stroke volume (Fig. 9 E and F). After 48 hours, all the different variables had returned to values comparable to those of control animals.

Most importantly, flagellin provoked direct negative inotropic effects, as evidenced by reductions of end-systolic elastance (Ees)-the slope of the LV end-systolic PV relationship, (Fig. 9G, with representative PV loops in the 4 groups of animals depicted in Fig. 9I), and of time-varying elastance, or maximal elastance, Emax (Fig. 9H). These changes, noted after 4 and 24 hours were no more observable at 48 hours.

## Discussion

The main characteristics of septic cardiomyopathy are depressed contractility, reduced LV ejection fraction and bi-ventricular dilation [7]. Recent studies have indicated that *intrinsic* alterations within cardiac myocytes are key mechanisms of this acute heart failure, which may be mediated by cardiac TLRs [8]. Cardiac myocytes express several TLRs [8], and several TLR ligands -LPS, peptidoglycan and bacterial DNA- activate NF-κB in these cells, respectively through activation of TLR4, TLR2 and TLR9, leading to the production of inflammatory mediators and alterations in normal cardiac physiology [9], [24], [25]. In the present study, we extend further this emerging concept, by showing that bacterial flagellin, the ligand of mammalian TLR5 [13], [14], [17], is a potent inducer of cardiac innate immune responses and contractile dysfunction.

Our results are the first to formally demonstrate that the heart expresses TLR5. Although the presence of TLR5 mRNA had been previously reported in the HL-1 cardiac cell line [26], we now show that TLR5 protein is expressed in vitro both by H9c2 cells and primary ventricular myocytes, and that it is also strongly expressed in cardiac tissue. Cardiac TLR5 expression was not restricted to mice and rats, but was also observed in humans, indicating that the ability of the heart to detect flagellin is not species-related.

In vitro, minute concentrations of flagellin activated NF-κB in H9c2 cells and primary cardiomyocytes, substantiating the presence of an intact TLR5-dependent signaling system in cardiac myocytes. The essential role of TLR5 in the process of NF-κB activation elicited by flagellin was clearly evidenced by the lack of NF-κB activation in primary murine cardiomyocytes isolated from neonatal TLR5 deficient mice. Flagellin also activated two major stress activated kinases, p38 and JNK, which may play a role in septic cardiomyopathy, notably by activating the production of TNFα within the myocardium [27], [28]. Taken together, these data indicate that recognition of extracellular flagellin by cells from cardiac origin triggers signaling cascades intimately linked with the development of cardiac dysfunction in sepsis.

In line with these in vitro results, flagellin also provoked major inflammatory changes in the heart in vivo, characterized by a robust activation of NF-κB, and the upregulation of the inflammatory cytokines TNFα, IL-1β, IL-6, MIP-2 and MCP-1 within the cardiac tissue. In accordance with the roles of MIP-2 and MCP-1 as chemokines attracting polymorphonuclear cells (PMN), respectively monocytes/macrophages, we found some recruitment of poly- and mononuclear cells in the heart after flagellin administration. The degree of activation of these infiltrating cells appeared, however, limited, given the modest increase in myeloperoxydase activity, and the lack of increase of cardiac TREM-1, a specific cytokine derived from myeloid cells [23], suggesting that the cardiac inflammatory phenotype triggered by flagellin did not result primarily from the activation of infiltrating inflammatory cells.

It must be underscored that NF-κB was activated extremely early upon the injection of flagellin, as shown by stimulated DNA-binding activity starting 10 minutes after the injection of flagellin. Such immediate response supports the hypothesis that cardiac inflammation was a direct consequence of flagellin-heart interactions rather than an indirect effect due to the remote generation of inflammatory mediators. Furthermore, NF-κB activation occurred at very low concentrations of flagellin, as indicated by a threshold-inducing dose of 10 ng/mouse (0.4 µg/kg), which are clinically relevant, given that plasma levels of free circulating flagellin between 2 and 16 µg/l have been detected in humans with septic shock [17]. Overall, these findings indicate that the heart is equipped to immediately recognize circulating flagellin with high sensitivity, implying that this bacterial protein may represent a particularly critical danger signal in this organ.

It is currently assumed that the local upregulation, within the heart itself, of inflammatory cytokines with negative inotropic properties, including TNFα, IL-1β [6], and IL-6, [29], as well as the cardiac recruitment of inflammatory cells, mostly polymorphonuclear cells [30], are key mechanisms leading to cardiac dysfunction in sepsis. The strong immune response of the heart in response to flagellin prompted us therefore to evaluate whether it would also affect cardiac physiology. To address this question, a microtip pressure volume (PV) conductance catheter was inserted into the left ventricle (LV) of mice, 4, 24 or 48 hours after an iv challenge with 1 µg (40 µg/kg) flagellin. In contrast to other methods, which provide indirect, load-dependent, indices of contractility, PV catheters allow the direct assessment of contractility by computing the slope of the LV end-systolic PV relationship (ESPVR), known as end-systolic elastance (Ees), which is independent from preload and afterload [31].

We could thus demonstrate that flagellin promotes the depression of cardiac contractility, as evidenced by cardiac dilation and reduced ejection fraction. These alterations were related to a direct negative inotropic effect, attested by significant reductions of both Ees and time-varying elastance, Emax. An important aspect of the cardiac dysfunction provoked by flagellin was its reversibility, as indicated by maximal systolic depression at 4 hours, followed by progressive recovery over a 48 hours period, a pattern which is highly consistent with the transient nature of septic myocardial dysfunction reported both in humans and in large aninal models of sepsis [5], [32]. Such reversibility argues against the development of significant cardiac damage upon flagellin administration. Indeed, we could not detect caspase activation in the heart and we did not notice any increase in plasma troponin I after flagellin, while systolic function was already significantly altered. This is in agreement with the lack of significant myocardial necrosis or apoptosis reported in animal models and in postmortem studies in septic shock patients, indicating that myocardial cell death is insufficient to account for the functional depression observed, as recently reviewed [6].

Although flagellin clearly depressed myocardial contractile function, our findings do not allow the conclusion that flagellin may be the causal factor in the development of cardiac dysfunction in sepsis. As stated earlier, many other factors released by bacteria may also promote myocardial inflammation and cardiac dysfunction, including bacterial DNA, lipopolysacharride and peptidoglycan. It is therefore highly likely that the depression of contractile function during sepsis is the result of the concerted action of multiple microbial virulence factors, each promoting intracellular innate immune defense mechanisms by interacting with distinct TLRs. To further precise the role of flagellin in the cardiac depression of bacterial sepsis would therefore require additional experiments using whole cell bacteria in TLR5 deficient mice, which should be performed in future studies.

Several limitations of our study deserve further discussion. Firstly, we cannot rule out that cells from non myocyte origin, mostly vascular endothelial and smooth muscle cells, as well as cardiac fibroblasts, may have contributed to the observed effects of flagellin in vivo. Secondly, our study may be criticized due to the lack of experiments in TLR5-deficient mice. However, these animals being unresponsive to flagellin [16], they would not allow to get further insights into the effects of flagellin on the heart. Cardiomyocyte-specific TLR5-knockout mice should therefore be developed in the future to further precise the actions of flagellin on this subset of cardiac cells. Thirdly, our findings do not prove that the depression of cardiac contractility observed after flagellin was a direct consequence of the cardiac immune response to flagellin. Indeed, the innate immune response to flagellin was not restricted to the heart, but instead was a widespread phenomenon, as shown by the rise of cytokines in other organs such as the lung and liver as well as the increase of circulating inflammatory mediators. Therefore, the contractile dysfunction observed upon flagellin administration cannot be simply attributed to a purely localized, cardiac restricted inflammation, but should be viewed as one component of the systemic innate immune response to this bacterial protein. In conclusion, our results collectively indicate that bacterial flagellin induces a prototypical innate immune response in cells from cardiac origin in vitro and in the whole heart in vivo, and that this response is associated with the development of an acute, transient and reversible, contractile dysfunction, which resembles the clinical picture of septic myocardial dysfunction.

## Materials and Methods

### Ethics Statement

All procedures involving animals complied with the Swiss laws on animal experimentation and were authorized by our institutional ethical committee for animal research (Veterinary Office of the State of Vaud, Department of Economy-Authorization Number 1827.0). The sampling of cardiac tissue from human patients at the time of cardiac surgery was approved by the ethical committee of the University of Lausanne, and written-informed consent was obtained from patients.

### Cell culture conditions and cell stimulations

H9c2 cells, a clonal cell line derived from rat heart retaining many biochemical and physiological aspects of adult rat cardiocytes [33] were grown as mentioned [22]. Key experiments were also performed in primary adult rat ventricular myocytes, isolated according to our published procedures [34], and also in primary cardiomyocytes obtained from neonatal wild-type and TLR5 deficient mice [35], according to previously published procedures [36]. Briefly, ventricles were separated from the atria, minced and digested in buffer containing 0.45 mg/ml collagenase (Worthington Biochemical Corporation) and 1 mg/ml pancreatin (Invitrogen Life Technologies). Cardiomyocytes were separated from non myocyte cells by two steps of differential plating at 45 min each, and were then plated on gelatinized plates in a 3∶1 mixture of DMEM and Medium 199 (Invitrogen life Technologies) supplemented with 10% horse serum (Serotech Ltd, Oxford, UK), 5% fetal bovine serum (FBS) (Serotech Ltd), 100 U/ml penicillin G, and 100 g/ml streptomycin. Cardiomyocytes were allowed to adhere overnight, and then maintained in serum-free medium with antibiotics before their stimulation with flagellin.

Cells were stimulated with 1–500 ng/ml *Salmonella muenchen* flagellin (Calbiochem, San Diego, CA), or 50–1000 ng/ml *Salmonella enteritidis* LPS (Sigma-Aldrich, Basel, Switzerland). In some experiments, flagellin or LPS were incubated for 10 minutes with polymyxin B (10 µg/ml) before use. Wild-type and TLR5 KO cardiomyocytes were left unstimulated or were stimulated with flagellin at 50 ng/ml for 30 minutes.

### Administration of flagellin to conscious mice

Male BALB/c mice (23–26 g) were injected (tail vein) with flagellin (40 ng/kg to 200 µg/kg) or vehicle (0.2 ml saline). At selected time-points, mice were used for hemodynamic measurements or were sacrificed to obtain hearts for biochemical analyses.

### Cardiac tissue sampling in humans

Human right atrial samples were obtained at the time of right atrial canulation during heart surgery.

### Immunoblot analyses

Total and nuclear protein extracts were prepared as described [21], and membrane-associated proteins were obtained after ultracentrifugation. SDS-PAGE and immunoblotting was performed as mentioned [20] using the following antibodies: anti-IκBα, anti-phospho-IκBα, anti-NF-κB RelA/p65, anti-JNK1, anti-p38 (Santa-Cruz Biotechnology, Santa Cruz, CA), anti-phospho-JNK1/2, anti-phospho-p38, anti-caspase-3, anti-α-tubulin (Cell signaling, Beverly, MA) and anti-TLR5 (Biovision Inc., Mountain View, CA).

### Electromobility Shift Assay (EMSA)

EMSA was performed as described [20], with an NF-κB oligonucleotide probe (5′-GGCAGTTGAAGGGGACTTTCCCAGG-3′) or an AP-1 oligonucleotide probe (5′-GGCAGCGCTTGATGACTCAGCCGAAA-3′). Competition studies were performed by adding a 33-fold excess of specific unlabeled NF-κB probe or mutated NF-κB probe (5′-GGCAGTTGAAGGCGACTTTCCCAGG-3′).

### IKK kinase assay

IKK immunoprecipitation and kinase assay were performed as mentioned [21].

### NF-κB Luciferase Reporter Assay

Cells were transiently transfected with 1 µg of a multimeric NF-κB pGL2 luciferase vector and 0.1 µg of the *Renilla* pRL-TK vector using Lipofectamine, and luciferase activity measured as described [20].

### RNA Isolation and RT-PCR

RNA isolation and reverse transcription were performed as described [21]. The sequences of the primer pairs for TNFα were as mentioned [21], and for rat MIP-2 (sense, 5′-TTGTTGTGGCCAGTGAGCTGC-3′; antisense, 5′-ATCAGGT ACGATCCAGGCTTCC-3′). A kit of mouse/rat specific PCR primer pair was purchased for TLR5 (R&D Systems, Minneapolis, MN).

### Immunofluorescence studies

H9c2 cells were fixed, permeabilized with Triton X-100 0.1%, blocked with goat serum, and incubated with normal rabbit serum or a rabbit polyclonal anti-TLR5 (BioVision). Immunodetection was performed using a goat anti-rabbit IgG secondary antibody labeled with Oregon Green 488 (Invitrogen). Nuclei were counterstained with DAPI.

### Quantification of cytokine production

The levels of TNFα, IL-1β, IL-6, MIP-2, MCP-1, and TREM-1 (triggering receptor expressed on myeloid cells-1), were measured in whole heart tissue by ELISAs (R&D Systems). MIP-2 was also quantified in the supernatant of H9c2 cells. In addition, the levels of TNFα, IL-1β and MIP-2 were also determined in liver and lung extracts as well as in the plasma 1 h after the administration of flagellin.

### Myeloperoxidase Assay

MPO activity was determined in cardiac tissue as described [22].

### Histology and immunohistochemistry of the heart

Hearts were fixed in 4% paraformaldehyde, embedded in paraffin, cut in 5 µm slice and stained with hematoxylin-eosin. Deparaffinized sections were incubated (1 h) in 5% rabbit serum, and then overnight at 4°C with rat anti-mouse Mac-3 immunoglobulin G1 at a 1/100 dilution (Pharmingen, BD Biosciences, San Diego, CA), which reacts with the 110-kDa Mac-3 surface antigen of mouse mononuclear phagocytes [37], followed by incubation for 40 min with a rabbit anti-rat secondary antibody conjugated to biotin (1/200, Vector, Burlingame, CA) and by 30 min incubation with DAB kit (Vector).

### Myocardial ischemia-reperfusion in rats

Myocardial ischemia was induced by transient (30 minutes) ligation of the left anterior descending coronary artery, followed by 3 hours reperfusion, in anesthetized, mechanically ventilated rats, according to our published procedures [22].

### Hemodynamic measurements in mice

The experiments were performed 4, 24 and 48 hours after flagellin (40 µg/kg, 1 µg/mouse) administration. The animals were anesthetized (ketamine, 75 mg/kg; xylazine, 10 mg/kg) and tracheotomized. A pressure-volume (PV) catheter (SPR-839; Millar Instruments) was inserted into the left ventricle (LV) via the right carotid artery. After stabilization for 20 minutes, heart rate, LV systolic and end-diastolic pressures and volumes were measured, and stroke volume, ejection fraction, and cardiac output were calculated and corrected according to in vitro and in vivo volume calibrations with a cardiac PV analysis program (PVAN3.2; Millar Instruments). End-systolic LV PV relationships were assessed by transiently reducing venous return by compressing the inferior vena cava, and LV contractility was assessed from the slope of the LV end-systolic PV relationship (end-systolic elastance), calculated using PVAN3.2, as detailed previously [38].

### Presentation of data and statistical analysis

All graphs summarize the results of at least 3 independent experiments, and are presented as means ± SEM. In experiments comparing only 2 conditions, statistical analysis was done with Student's *t*-test. In experiments using multiple conditions, comparison was done with analysis of variance (ANOVA) followed by Dunnett post-hoc test when appropriate. A *p* value<0.05 was considered significant.

## References

[1] Russell JA (2006). Management of sepsis.. N Engl J Med.

[2] Trinchieri G, Sher A (2007). Cooperation of Toll-like receptor signals in innate immune defence.. Nat Rev Immunol.

[3] Dowling D, Hamilton CM, O'Neill SM (2008). A comparative analysis of cytokine responses, cell surface marker expression and MAPKs in DCs matured with LPS compared with a panel of TLR ligands.. Cytokine.

[4] Zhang Z, Reenstra W, Weiner DJ, Louboutin JP, Wilson JM (2007). The p38 mitogen-activated protein kinase signaling pathway is coupled to Toll-like receptor 5 to mediate gene regulation in response to Pseudomonas aeruginosa infection in human airway epithelial cells.. Infect Immun.

[5] Parker MM, Shelhamer JH, Bacharach SL, Green MV, Natanson C (1984). Profound but reversible myocardial depression in patients with septic shock.. Ann Intern Med.

[6] Rudiger A, Singer M (2007). Mechanisms of sepsis-induced cardiac dysfunction.. Crit Care Med.

[7] Merx MW, Weber C (2007). Sepsis and the heart.. Circulation.

[8] Flierl MA, Rittirsch D, Huber-Lang MS, Sarma JV, Ward PA (2008). Molecular events in the cardiomyopathy of sepsis.. Mol Med.

[9] Knuefermann P, Nemoto S, Misra A, Nozaki N, Defreitas G (2002). CD14-deficient mice are protected against lipopolysaccharide-induced cardiac inflammation and left ventricular dysfunction.. Circulation.

[10] Nemoto S, Vallejo JG, Knuefermann P, Misra A, Defreitas G (2002). Escherichia coli LPS-induced LV dysfunction: role of toll-like receptor-4 in the adult heart.. Am J Physiol Heart Circ Physiol.

[11] Constable PD (1999). Acute endotoxemia increases left ventricular contractility and diastolic stiffness in calves.. Shock.

[12] Pinsky MR, Rico P (2000). Cardiac contractility is not depressed in early canine endotoxic shock.. Am J Respir Crit Care Med.

[13] Eaves-Pyles T, Murthy K, Liaudet L, Virag L, Ross G (2001). Flagellin, a novel mediator of Salmonella-induced epithelial activation and systemic inflammation: I kappa B alpha degradation, induction of nitric oxide synthase, induction of proinflammatory mediators, and cardiovascular dysfunction.. J Immunol.

[14] Hayashi F, Smith KD, Ozinsky A, Hawn TR, Yi EC (2001). The innate immune response to bacterial flagellin is mediated by Toll-like receptor 5.. Nature.

[15] Andersen-Nissen E, Hawn TR, Smith KD, Nachman A, Lampano AE (2007). Cutting edge: Tlr5-/- mice are more susceptible to Escherichia coli urinary tract infection.. J Immunol.

[16] Feuillet V, Medjane S, Mondor I, Demaria O, Pagni PP (2006). Involvement of Toll-like receptor 5 in the recognition of flagellated bacteria.. Proc Natl Acad Sci U S A.

[17] Liaudet L, Murthy KG, Mabley JG, Pacher P, Soriano FG (2002). Comparison of inflammation, organ damage, and oxidant stress induced by Salmonella enterica serovar Muenchen flagellin and serovar Enteritidis lipopolysaccharide.. Infect Immun.

[18] Liaudet L, Szabo C, Evgenov OV, Murthy KG, Pacher P (2003). Flagellin from gram-negative bacteria is a potent mediator of acute pulmonary inflammation in sepsis.. Shock.

[19] Gewirtz AT, Navas TA, Lyons S, Godowski PJ, Madara JL (2001). Cutting edge: bacterial flagellin activates basolaterally expressed TLR5 to induce epithelial proinflammatory gene expression.. J Immunol.

[20] Loukili N, Rosenblatt-Velin N, Rolli J, Levrand S, Feihl F (2010). Oxidants positively or negatively regulate nuclear factor kappa B in a context-dependent manner.. J Biol Chem.

[21] Levrand S, Pesse B, Feihl F, Waeber B, Pacher P (2005). Peroxynitrite is a potent inhibitor of NF-{kappa}B activation triggered by inflammatory stimuli in cardiac and endothelial cell lines.. J Biol Chem.

[22] Levrand S, Vannay-Bouchiche C, Pesse B, Pacher P, Feihl F (2006). Peroxynitrite is a major trigger of cardiomyocyte apoptosis in vitro and in vivo.. Free Radic Biol Med.

[23] Murakami Y, Kohsaka H, Kitasato H, Akahoshi T (2007). Lipopolysaccharide-induced up-regulation of triggering receptor expressed on myeloid cells-1 expression on macrophages is regulated by endogenous prostaglandin E2.. J Immunol.

[24] Knuefermann P, Sakata Y, Baker JS, Huang CH, Sekiguchi K (2004). Toll-like receptor 2 mediates Staphylococcus aureus-induced myocardial dysfunction and cytokine production in the heart.. Circulation.

[25] Knuefermann P, Schwederski M, Velten M, Krings P, Ehrentraut H (2008). Bacterial DNA induces myocardial inflammation and reduces cardiomyocyte contractility: role of Toll-like receptor 9.. Cardiovasc Res.

[26] Boyd JH, Mathur S, Wang Y, Bateman RM, Walley KR (2006). Toll-like receptor stimulation in cardiomyoctes decreases contractility and initiates an NF-kappaB dependent inflammatory response.. Cardiovasc Res.

[27] Peng T, Lu X, Lei M, Moe GW, Feng Q (2003). Inhibition of p38 MAPK decreases myocardial TNF-alpha expression and improves myocardial function and survival in endotoxemia.. Cardiovasc Res.

[28] Yang M, Wu J, Martin CM, Kvietys PR, Rui T (2008). Important role of p38 MAP kinase/NF-{kappa}B signaling pathway in the sepsis-induced conversion of cardiac myocytes to a proinflammatory phenotype.. Am J Physiol Heart Circ Physiol.

[29] Zhang H, Wang HY, Bassel-Duby R, Maass DL, Johnston WE (2007). Role of interleukin-6 in cardiac inflammation and dysfunction after burn complicated by sepsis.. Am J Physiol Heart Circ Physiol.

[30] Neviere RR, Cepinskas G, Madorin WS, Hoque N, Karmazyn M (1999). LPS pretreatment ameliorates peritonitis-induced myocardial inflammation and dysfunction: role of myocytes.. Am J Physiol.

[31] Suga H, Sagawa K (1974). Instantaneous pressure-volume relationships and their ratio in the excised, supported canine left ventricle.. Circ Res.

[32] Natanson C, Fink MP, Ballantyne HK, MacVittie TJ, Conklin JJ (1986). Gram-negative bacteremia produces both severe systolic and diastolic cardiac dysfunction in a canine model that simulates human septic shock.. J Clin Invest.

[33] Hescheler J, Meyer R, Plant S, Krautwurst D, Rosenthal W (1991). Morphological, biochemical, and electrophysiological characterization of a clonal cell (H9c2) line from rat heart.. Circ Res.

[34] Levrand S, Pacher P, Pesse B, Rolli J, Feihl F (2007). Homocysteine induces cell death in H9C2 cardiomyocytes through the generation of peroxynitrite.. Biochem Biophys Res Commun.

[35] Uematsu S, Jang MH, Chevrier N, Guo Z, Kumagai Y (2006). Detection of pathogenic intestinal bacteria by Toll-like receptor 5 on intestinal CD11c+ lamina propria cells.. Nat Immunol.

[36] Rosenblatt-Velin N, Lepore MG, Cartoni C, Beermann F, Pedrazzini T (2005). FGF-2 controls the differentiation of resident cardiac precursors into functional cardiomyocytes.. J Clin Invest.

[37] Waeckel L, Bignon J, Liu JM, Markovits D, Ebrahimian TG (2006). Tetrapeptide AcSDKP induces postischemic neovascularization through monocyte chemoattractant protein-1 signaling.. Arterioscler Thromb Vasc Biol.

[38] Pacher P, Liaudet L, Bai P, Mabley JG, Kaminski PM (2003). Potent metalloporphyrin peroxynitrite decomposition catalyst protects against the development of doxorubicin-induced cardiac dysfunction.. Circulation.

